# Experimental Analysis of Moisture-Dependent Thermal Conductivity, and Hygric Properties of Novel Hemp–shive Insulations with Numerical Assessment of Their In-Built Hygrothermal and Energy Performance

**DOI:** 10.3390/ma17020486

**Published:** 2024-01-19

**Authors:** Eshrar Latif

**Affiliations:** Welsh School of Architecture, Cardiff University, Cardiff CF10 3NB, UK; latife@cardiff.ac.uk

**Keywords:** hemp–shive insulation, thermal conductivity, adsorption isotherm, vapour resistance, water absorption coefficient, energy use, thermal comfort, bio-based binder, hygrothermal performance, bio-based insulation

## Abstract

The use of lime as a binder in hemp–lime considerably increases the drying time of hemp–lime after casting. Furthermore, lime is a non-renewable mineral resource. As such, this paper explores the effectiveness of using an alternative non-mineral binder instead of lime to formulate a novel hemp–shive insulation. The moisture-dependent thermal conductivity, adsorption isotherm, vapour diffusion resistance factor, and in-built hygrothermal performance of four variants of a novel bio-based insulation were investigated. The hygrothermal performance of the novel hemp–shive insulation was compared with that of a previously developed novel hemp–lime insulation. No significant variation in thermal conductivity of hemp–shive insulations between the equilibrium moisture contents (EMC) at 0% and 50% relative humidity (RH) was observed, but there was a substantial increase in thermal conductivity hemp–shive insulations when the material reached the EMC at 98% RH. The average dry thermal conductivity values of hemp–shive and hemp–lime insulations were also similar. The adsorption isotherms of hemp–shive insulations were determined at 0%, 20%, 50%, 70%, 90%, and 98% relative humidity steps. At 98% RH, the moisture adsorption capacity of hemp–shive insulations was 4-to-5-times higher than that of hemp–lime insulation. Hemp–shive insulations’ vapour diffusion resistance factor (µ value) was about double the µ value of hemp–lime insulation. Hemp–shive insulations exhibited 4-to-5-times higher water absorption resistance than that of hemp–lime insulation. Numerically determined porosity values of hemp–shive agree with the values of wood-based insulation materials of similar density. Finally, using all experimentally acquired data as inputs, dynamic whole-building hygrothermal simulations were carried out and the results show that novel hemp–shive insulation materials perform at a similar level to the hemp–lime insulation in terms of heating and cooling energy demand but require 45% less energy for humidification. However, the relative humidity inside the hemp–shive wall remains higher than 70%, which can potentially induce mould growth.

## 1. Introduction

The evolving net-zero building standard in the UK is considering taking embodied carbon into account in addition to aiming at benchmarking the operational energy demand [1]. While the concept of circular economy emphasises maintaining, reducing, reusing, and recycling of materials [2,3], circularity at the end of life after multiple life cycles is also a concern. For the concept of circular economy to be coupled with the future net-zero standard, building materials should fully comply with the concept of sustainability. A sustainable material is expected to be of low embodied energy, manufactured from renewable materials, performing at least to the same level as a conventional material for any intended use, and be biodegradable after the active life cycles. Buildings are responsible for 40% of European Union energy consumption and 36% of greenhouse gas emissions [4]. In the UK, the domestic sector consumed 31% of the total energy in 2021 [5], and about 60% of the total domestic energy is used for space heating [6]. Since building envelope is the first line of defence against the elements of weather, for the UK to achieve net-zero energy by 2050, the existing buildings need to be thermally refurbished, and the new buildings should have highly insulative envelopes without neglecting the mechanisms of mitigating summer overheating by careful application of thermal mass. Another emergent problem in the built environment is the effect of poor indoor air quality (IAQ) on vulnerable people. In addition to the influence of external air-born pollutants, poor IAQ can be the consequence of attaining highly energy efficient air-tight buildings, emissions of volatile organic compounds (VOCs) such as formaldehyde from building materials, emissions of biological pollutants like mould spores from interior surfaces, and particulate matters (PMs) generation through cooking and combustion activities.

Bio-based building materials possess moderate-to-high thermal insulative properties, high heat capacity, high moisture buffer value, lower-to-medium embodied carbon, and some of them adsorb certain VOCs [7]. Thus, bio-based building materials can potentially address the issues of circular economy in practice, achieving high hygrothermal performance and maintaining good indoor air quality. Like conventional building materials, bio-based materials can also be applied to roofs, walls, and floors. Some of the key bio-based insulation materials are sheep wool insulation, straw, wood fibre insulation, flax fibre insulation, hemp fibre insulation, and hemp–lime insulations. Among these materials, hemp-fibre and hemp–lime insulations have gained momentum in the construction industry in recent years due to a number of factors. Industrial hemp, containing a very low level of delta-9 THC (tetrahydrocannabinol), has a very high annual yield of 7–9 tonnes per hectare. Hemp crop suppresses weeds, and requires minimal biocide and a very limited amount of fertiliser [8]. The stem of hemp consists of wood and bast tissue. Hemp fibre insulation is manufactured from the bast fibres while hemp–lime insulation is manufactured from the woody core (hemp–shive) mixed with lime binder. In terms of embodied carbon, lime is not much different from cement [9,10] and, as such, research has been carried out in recent years to replace lime binder with more sustainable bio-based binders.

There is a substantial number of research articles that have been published on the hygrothermal, fire, and mechanical performance of hemp–lime insulation [11,12,13,14,15,16,17] indicating that hemp–lime, comprising of hemp–shive and lime binder, demonstrates moderate thermal conductivity ranging from 0.06 to 0.12 W/m.K, with specific heat capacity ranging between 1300 and 1700 J/kg.K and a density usually ranging between 220 and 950 kg/m^3^. Furthermore, hemp–lime possesses excellent moisture buffering capacity and can be moulded into various rigid forms and utilised as flooring, walling, or roofing materials. However, one of the major problems with hemp–lime is that a high quantity of water is required during the mixing of hemp and lime as both compete for water. It results in a prolonged drying up time of 3 to 6 months for the hemp–lime envelope after casting. During the drying up period, the hemp–lime envelope performs poorly in terms of thermal and surface mould resistance, necessitating the search for an alternative binder.

A limited number of research papers have explored the use of alternative binders in hemp–lime with lower carbon impacts [18,19,20]. Several industrial and academic researchers have explored the possibilities of developing bio-based binders for various applications, including the application in non-woven products. Bragagila et al. successfully used polylactic acid as bio-based binder for the production of 3D-printing filaments for Ti6Al4V alloy manufacturing via bound metal deposition [21]. Wennman et al. described the use of a polyelectrolyte complex as a combined emulsifier and carrier of hydrophobic substances to cellulosic surfaces [22]. Wennman et al. further developed a sustainable binder based on wheat gluten (WG) and a polyelectrolyte complex (PEC) made from chitosan, carboxymethyl cellulose, and citric acid, which can be used with cellulosic fibres, creating a fully bio-based nonwoven product [23]. The potential of environmentally friendly bio-based polyfurfuryl alcohol resin is discussed by Odiyi et al. [24]. Chemical modification of lignin to produce bio-based binders has been explored by a number of researchers [25,26].

Currently, a novel bio-based hemp–shive insulation product is under development using mineral or non-mineral liquid sodium silicate with or without tributyl ester. The embodied energy of conventional sodium silicate can vary between 4.6 and 17.9 MJ/kg, with most papers reporting a value of 4–5 MJ/kg [27,28,29] compared to 3–5.3 MJ/kg embodied energy of lime [30,31]. However, liquid sodium silicate has other properties that can plausibly outperform lime as a binder for hemp-based insulations. Firstly, sodium silicate is also being bio-derived from rise husk ash [32] and is commercially available. Secondly, the mass ratio of binder to hemp–shive can be substantially reduced, resulting in a lower overall embodied energy in the insulation. Furthermore, since high water absorption seems to be disadvantageous during the production and casting of hemp–shive-based insulation, it is expected that the sodium silicate with tributyl ester will increase the liquid water absorption resistance of hemp–shive. The current paper limits its aims to assess the thermal, hygric. and in-built energy performance potential of the newly developed bio-based hemp–shive insulation materials. Additionally, the paper also compares the new insulations’ hygrothermal performance with a novel hemp–lime insulation that was developed in the Hempsec [33] project. While the importance of fire protection and smart sensing of fire risk is very important for thermal insulation materials, this is subject to a separate investigation [34].

## 2. Materials and Methods

### 2.1. Material

**Figure 1:**
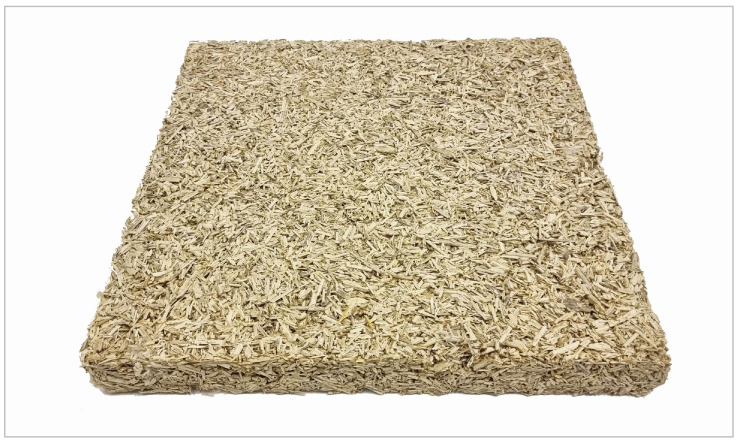
A sample hemp–shive insulation.

Four variants of innovative hemp–shive insulation were developed. Each type (Figure 1) was prepared by mixing hemp–shive with the non-mineral liquid sodium silicate binder in a planetary mixer for five minutes so that the shives were evenly covered by the binder. The length of the shives varied between 6 mm and 22 mm, with an average shive length of 10 mm and an average aspect ratio of 2.9. Moulds with inner dimension of 150 mm × 150 mm × 50 mm (length × width × height) were filled with the composite, ensuring that the corners are filled and the composite is lightly pressed from the top. Afterwards, the top surface was smoothed to make it flat. The samples were then left for 18 h to set and then dried on a ventilated worktop with an air flow rate of 1 m/s at room temperature for 24 h. The dimension, the bulk density at dry state, and constituent ratios in the samples are provided in Table 1. The difference between A1 and A2 was the addition of 10% tributyl ester to the binder in A2. The difference between B1 and B2 was the same with 10% tributyl ester to the binder in B2.

### 2.2. Method

As part of the hygrothermal characterisation, density, dry thermal conductivity, moisture-dependent thermal conductivity, adsorption isotherm, vapour diffusion resistance factor, porosity, and water absorption coefficient values of the biocomposite insulations were determined following international and British standards, as applicable. Finally, the in-built hygrothermal and energy performance of two identical buildings with walls composed of hemp–shive and hemp–lime insulations was compared using a whole-building hygrothermal simulation tool.

#### 2.2.1. Determination of Adsorption Isotherm

The adsorption isotherms of hemp–shive insulations were determined following British Standard BS EN ISO 12571 [35]. To determine the adsorption isotherm, three samples of each material type with dimensions of 150 mm × 150 mm × 50 mm were exposed to five increasing humidity levels ranging from 0% to 98% while maintaining a constant temperature of 23 (±0.5) °C in a climate chamber. During each exposure, the samples had to reach equilibrium moisture content (EMC). EMC was considered to have been achieved when the change in mass between three consecutive weighing, each separated by at least 24 h, was less than 0.1% of the total mass. The calculation for the weight-based moisture content adsorption (u), expressed in kilograms per kilogram (kg/kg), is as follows:

u = (m − m_0_)/m_0_ (1)

where: 

m_0_ represents the material’s mass under dry conditions (measured in grams, g).

m represents the material’s mass at equilibrium moisture content at any given relative humidity (measured in grams, g).

#### 2.2.2. Thermal Conductivity

Thermal conductivity by steady-state method: The Fox 600 Heat Flow meter (TA Instruments, Cheshire, UK) measures the thermal conductivity of large samples according to ASTM C518 [36] and ISO 8301 [37] when a temperature gradient is established over the thickness of the material. The following equations were used to determine thermal conductivity:

λ = U × d
(2)

where

λ is thermal conductivity (W/m.K)

U is thermal transmittance (W/m^2^.K)

d is the depth of the sample (m)

R = 1/U = (T_1_ − T_2_)/(f × е_h_) (3)

where: f is the calibration factor of the heat flow meter; e_h_ is the heat flow meter output; T_1_ and T_2_ are the temperatures at hot and cold sides.

The Fox 600 Heat Flow Meter was used to determine the steady-state thermal conductivity of the samples with an average dimension of (400 × 400 × 50) mm.

Thermal conductivity by transient method: The measurement, by an Isomet 2114 Thermal Properties Analyzer (Applied Precisions Ltd., Rača, Slovakia), is based on the thermal response of the sample to the induced heat flow. The materials’ temperature as a function of time was analysed to determine thermal conductivity. The following equations based on ASTM D 5334-08 [38] was used for determining thermal conductivity:

ΔT = [Q/(4πλ)] × [ln(t_2_/t_1_)] (4)

where

ΔT is the temperature increase from time zero (K);

Q is the heat input per unit length of heater (W/m);

λ is the thermal conductivity (W/m.K);

t_1_ is the initial time;

t_2_ is the final time.

For the current research, the thermal conductivities of three samples of each material type with a nominal dimension of (150 × 150 × 50) mm were determined. Each determination consisted of an average thermal conductivity value of two-to-three runs, and the thermal conductivity of 3 samples of each insulation type was measured. The materials were kept at 50% relative humidity and 23 °C temperature for three months prior to the measurements.

Moisture-dependent thermal conductivity calculation: 

The thermal conductivity of hemp–shive insulations was measured at different equilibrium moisture contents (EMC) at 0%, 50%, and 98% relative humidity (RH). EMCs were attained by exposing the materials to the target relative humidity in a climate chamber at 23 °C temperatures until a constant mass was obtained. The material was then covered with a thin polyethylene film so that there was no moisture loss, and the thermal resistance of the film was taken into account during the calculation. Furthermore, each sample as placed in an enclosure, and transient thermal conductivity value was determined with a disk probe.

In addition to the determination of moisture-dependent thermal conductivity by experiments, mathematical equations were also applied to determine the moisture conversion coefficient mass by mass (f_u_) for the conversions of thermal conductivity at from 0% to 98% relative humidity at 23 °C temperature, and this was also determined for each insulations using the following two equations:

λ_2_ = λ_1_F_T_ F_m_ F_a_ (5)

where

λ_1_ and λ_2_ are initial and changed thermal conductivity values, respectively.

F_T_ is the conversion factor for temperature. 

F_m_ is the conversion factor for moisture.

F_a_ is the conversion factor for age.

Since the current focus is only moisture, F_T_ and F_a_ can be ignored and the equation can be written as:

λ_2_ = λ_1_ F_m_ (6)

and F_m_ can be determined by mass or volume. 

For conversion of moisture content given as mass by mass:

F_m_ = e^f^_u_^(u^_2_^− u^_1_^)^ (7)

where

e = 2.71828183;

f_u_ is the moisture conversion coefficient mass by mass;

u_1_ is the moisture content mass by mass of the first set of conditions;

u_2_ is the moisture content mass by mass of the second set of conditions.

For conversion of moisture content given as volume by volume:

F_m_ = e^f^_ψ_ ^(ψ^_2_^− ψ^_1_^)^ (8)

where

f_ψ_ is the moisture conversion coefficient volume by volume.

ψ_1_ is the moisture content volume by volume of the first set of conditions.

ψ_2_ is the moisture content volume by volume of the second set of conditions.

#### 2.2.3. Determination of Vapour Diffusion Resistance Factor

The dry cup vapour diffusion resistance factors of thermal insulating materials were determined following British Standard BS EN 12086 [39]. Three samples measuring 150 mm × 150 mm with a thickness of 50 mm were placed on glass dishes containing desiccants. The sides of the insulations were effectively sealed to enable one-directional moisture flow. The relative humidity inside the dishes was set at 0%, while outside the dishes was maintained at 50 (±3) % in a climate chamber. The assembly was weighed every 24 h until five successive determinations of the change in mass per unit time for each specimen fell within ±5% of the mean value. The rate of change in mass was calculated using the formula:

G_1,2_ = (m_2_ − m_1_)/(t_2_ − t_1_) (9)

where m_1_ (in milligrams, mg) represents the mass of the test assembly at time t_1_, m_2_ is the mass at time t_2_, and t_1_ and t_2_ are successive weighing times (in hours). G is the mean of five successive G_1,2_ determinations (in mg/h), and G_1,2_ is within ±5% of G. The vapor diffusion resistance factor, µ, was calculated as:

µ = δ_a_/δ (10)

#### 2.2.4. Determination of Water Absorption Coefficient

The British Standard BS EN 15148 [40] outlines the procedures for experimentally determining the water absorption coefficient (referred to as the A value) of building materials through partial immersion. The test specimens were conditioned to a temperature range of 18–28 °C, with a permissible temperature fluctuation of ±2 °C, and a relative humidity range of 40% to 60%, with a permissible relative humidity variation of ±5. The samples were placed in tanks, supported on point supports to prevent direct contact with the tank surfaces. The tanks were filled with water to maintain the water level at (5 ± 2) mm above the highest point on the specimen’s base. The sides of the samples were sealed with a water- and vapor-tight sealant. All samples were weighed at specific time intervals, including 5 min, 20 min, 1 h, 2 h, 4 h, 8 h, 16 h, and 24 h. The difference in mass per unit area (W) between each weighing and the initial weighing was calculated using the formula:

W = (m_t_ − m_i_)/a (11)

where

m_t_ represents the mass at any time of weighing (in kilograms).

m_i_ represents the mass at the initial weighing (in kilograms)

a represents the surface area of the sample in contact with water (in square meters).

The water absorption coefficient (A value) can be expressed as A_w_, W_w_, A_w,24_, or W_w,24_. To determine the A value, Δm_t_ = m_t_ − m_i_ was plotted against the square root of the weighing times. When a linear relationship can be observed among the Δm_t_ values, the line is extended back to zero time, and the intercept’s value is denoted as Δm’_0_. In this context, the water absorption coefficient was represented as either A_w_ or W_w_, and it can be calculated using either of the following equations (Equations (12) and (13)):

A_w_ = (Δm′_tf_ − Δm′_0_)/√tf (12)

W_w_ = (Δm′_tf_ − Δm′_0_)/√tf (13)

where

Δm′_tf_ (in kg/m^2^) is the Δm value at time ‘tf’.

The time ‘tf’ is equivalent to 1 day expressed in seconds (in Equation (12)) or 1 day expressed in hours (in Equation (13)).

When the plot of Δm_t_ against the square root of the time does not yield a straight line, the water absorption coefficient is denoted as A_w,24_ or W_w,24_, and it can be calculated using either of the following equations (Equations (14) and (15)):

A_w,24_ = Δm′_tf_/√tf (14)

W_w,24_ = Δm′_tf_/√tf (15)

where:

Δm′_tf_ (in kg/m^2^) represents the Δm value at time ‘tf’.

The time ‘tf’ is equivalent to 1 day expressed in seconds (in Equation (14)) or 1 day expressed in hours (in Equation (15)).

#### 2.2.5. Calculation of Porosity

Porosity can be calculated using the following equation [41]:

Φ = [V_bulk_ − (W_dry_/ρ_matrix_)]/V_bulk_ (16)

where:

V_pore_ is the pore volume;

V_bulk_ is the bulk volume;

V_matrix_ = volume of solid particles composing the matrix;

W_dry_ = total dry weight;

ρ_matrix_ = mean density of the matrix minerals.

#### 2.2.6. Comparison of Energy Use in a Simplified Building following Bestest Configuration

**Figure 2:**
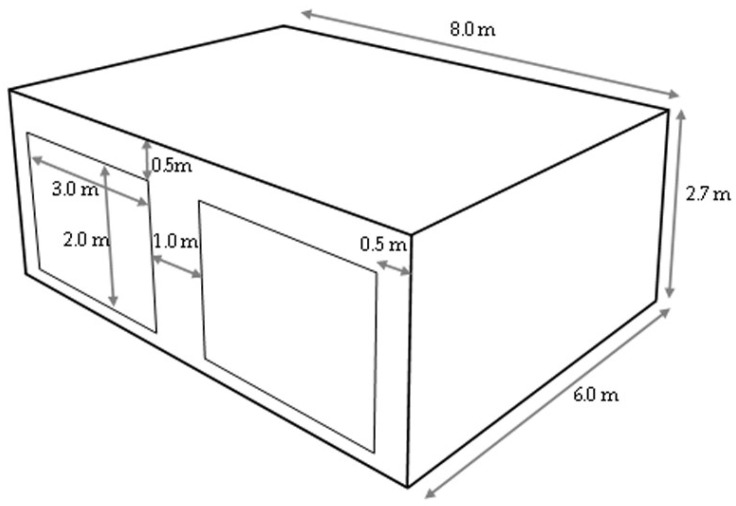
The geometric configuration of the computer simulation model.

Building dimension and openings: The primary aim of developing the innovative hemp–shive insulations was to create a variant of hemp–lime without using lime so that the embodied carbon of the material was reduced but the hygrothermal functionality and performance level remained similar to hemp–lime. As such, it was vital to compare the hygrothermal performance of both materials in a simplified building using a reliable hygrothermal simulation tool. The building dimension and openings (Figure 2) proposed by the Building Energy Simulation Test (Bestest) procedure [42] were followed to configure the simplified buildings so that the data could be easily compared.

Building Materials: The hemp–lime material data were collected from a number of research articles and academic books [11,43] that covered the ‘Hempsec’ project. The hemp–shive data were acquired in the current research by experimental methods.

Building and comfort parameters: The building envelope parameters and internal comfort settings are provided in Table 2 and Table 3., respectively. The building materials were initially exposed to 20 °C temperature and an EMC at 89% relative humidity. The building was equipped with a simplified HVAC system with 85% efficiency. The space heating and cooling capacity was 100 kW, and the humidification and dehumidification capacity was 100 kg/h.

Numerical hygrothermal simulation tool: 

WUFI Plus was selected as the numerical tool to simulate and compare the hygrothermal performance of the building with novel hemp–shive and hemp–lime insulations in the fabric. WUFI uses the following coupled heat and moisture balance equations, and the solutions were obtained by the finite volume method:

(17) $$\frac{dH}{dT}\frac{\partial T}{\partial t}=\nabla .\left(\lambda *\nabla T\right)+L_{lv}\nabla .\left(\delta p\nabla \left(\varphi \rho_{sat}\right)\right)$$

(18) $$\frac{dw}{d\varphi }\frac{\partial \varphi }{\partial t}=\nabla .\left(D\varphi \nabla \varphi +\delta p\nabla \left(\varphi \rho_{sat}\right)\right)\;$$

where,

$\frac{dH}{dT}\;$ = heat storage capacity of moist building material (J/m^3^K);

$\frac{dw}{d\varphi }$ = moisture storage capacity of building material (kg/m^3^);

λ* = thermal conductivity of moist material (W/m.K);

dφ = liquid conduction coefficient of building material (kg/ms);

Δp = Water vapour permeability of building material (kg/msPa);

$L_{lv}$ = evaporation enthalpy of water (J/kg);

Ρ_sat_ = saturation vapour pressure (Pa);

T = temperature (°C);

φ = relative humidity (-).

WUFI Plus has the capability to comprehensively simulate the dynamic hygrothermal behaviour of buildings and envelopes. Following experimentally acquired material, data were provided as input parameters: density, moisture-dependent thermal conductivity, vapour diffusion resistance factor, adsorption isotherm, porosity, specific heat capacity, and water absorption coefficient (Table 4).

In addition to the material data, WUFI Plus also requires external weather data that include rain data. For the purpose of this paper, the WUFI weather file of Brussels was selected from the WUFI database as Brussels is near to the UK and the weather data contain rain data. The envelope parameters and comfort condition are provided in Table 2 and Table 3. The simulation was run for one year.

#### 2.2.7. Sensors and Equipment

Following sensors and equipment are used during the experimental tests:

Climate chamber: A Hongjin programmable temperature and humidity climate chamber (Dongguan Hongjin Test Instrument Co., Ltd., Dongguan, China) was used for adsorption isotherm, vapour diffusion resistance factor and moisture-dependent thermal conductivity measurements. The climate chamber can generate a humidity range of from 20% to 98% with a precision of ±2.5% RH and a temperature range of from 0 °C to 150 °C with a precision of ±0.5 °C. 

Tempo Disc™ Wireless 4 in 1 Sensor and Logger (BlueMaestro, 10 York Rd, London, UK): This wireless sensor can measure from 0% to 100% relative humidity with ±1% accuracy, from −30 °C to +85 °C temperature with 0.3 °C accuracy, from 300 hPa to 1100 hPa barometric pressure (accuracy is not given), and has a dew point accuracy that is similar to the temperature one. The memory capacity is 6000 data points for each sensor type.

Tempo Disc™ Wireless Temperature, Humidity and Dew Point Sensor Beacon and Data Logger (BlueMaestro, 10 York Rd, London, UK): This wireless sensor can measure from 0% to 100% relative humidity with ±3% accuracy, from −30 °C to +75 °C temperature with 0.3 °C accuracy,) and has a dew point accuracy that is similar to the temperature one. And, the memory capacity is 6000 data points for each sensor type.

FOX 600 heat flow meter (TA Instruments, Cheshire, UK): The Fox 600 Heat Flow Meter measures thermal conductivity of large samples according to ASTM C518 and ISO 8301 standards. It can operate between −10 °C and 65 °C with a thermal conductivity accuracy of ±1%. Samples were positioned between two plates within the test stack, creating a temperature gradient across the material’s thickness. The plates can be adjusted to a user-specified thickness or set using Auto Thickness, where the instrument autonomously aligns with the sample. In situ sample thickness is gauged using four optical encoders, one situated at each plate’s corner, ensuring precise measurements with a margin of error of only 0.025 mm.

Isomet 2114: The Isomet 2114 is a portable system for the measurement of thermal conductivity, thermal diffusivity, volume heat capacity, and temperature using a transient method. A disk probe and a needle probe can be used for non-invasive and slightly invasive measurements, respectively.

Extech SDL350 Hotwire Anemometer Datalogger (Extech Instruments, Nashua, NH, USA): The Extech SDL350 Hot Wire Thermo-Anemometer Datalogger meter displays and stores 20 million air velocity and temperature readings. The measurement range is from 0.2 to 25 m/s, its resolution is 0.01 m/s, and its accuracy is ±5%.

Adam HCB 2202 precision weighing scale (Adam Equipment, Milton Keynes, UK): The Adam HCB 2202 precision weighing scale has a maximum weighing capacity of 2.2 kg, a resolution of 0.01 g, and a repeatability of 0.03 g.

## 3. Results and Discussion

### 3.1. Adsorption Isotherm

**Figure 3:**
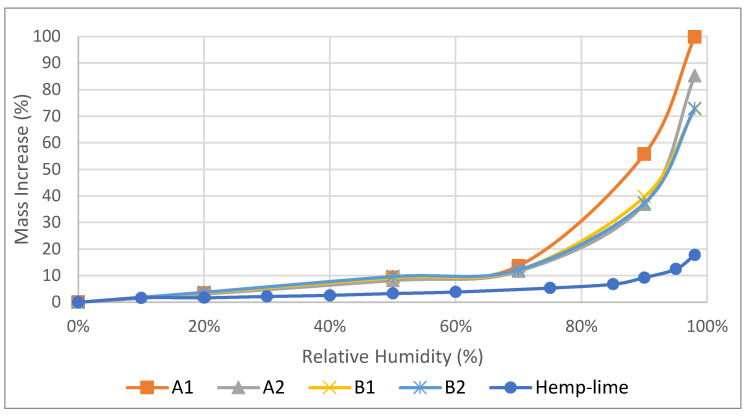
Adsorption isotherm of the samples A1, A2, B1, B2 by mass.

**Figure 4:**
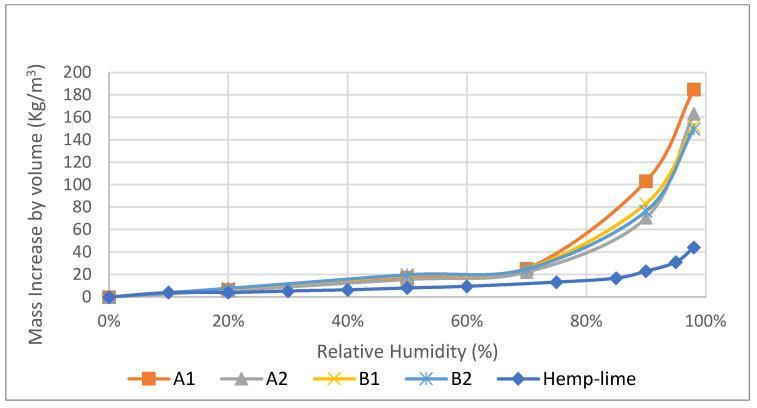
Adsorption isotherm of the samples A1, A2, B1, B2 by volume.

The adsorption isotherms of all hemp–shive insulations were determined in a programmable climate chamber. The adsorption isotherms (Figure 3 and Figure 4) follow the IUPAC Type II pattern, where unrestricted multilayer adsorption occurs at higher vapour pressure. This is an ideal characteristic for indoor moisture control by moisture buffering. A1 insulation exhibited the highest adsorption capacity at high relative humidity ranges. Nevertheless, all materials showed a similar adsorption capacity up to 70% relative humidity exposure at 23 °C temperature. It can be further observed that B1 and B2 absorbed less moisture at 98% relative humidity; at this EMC region, condensation occurs in micropores of hemp–shive. It seems that the higher binder ratios in B1 and B2 may have contributed to liquid water resistance.

All mass adsorption values were converted to volumetric adsorption capacities as volumetric adsorption capacity is a more practical measure of understanding moisture adsorption by materials in real life. When compared with hemp–lime composite, it is observed (Figure 3 and Figure 4) that, at a higher humidity range, the adsorption capacities both by mass and volume of novel hemp–shive insulations are three to five times higher than the adsorption capacity of hemp–lime composite [11] and closer to that of some hemp fibre insulations [44]. Due to the ability of the climate chamber to attain 98% relative humidity at 23 °C, an exponential increase in moisture content could be observed between 70% and 98% relative humidity ranges.

### 3.2. Thermal Conductivity

**Figure 5:**
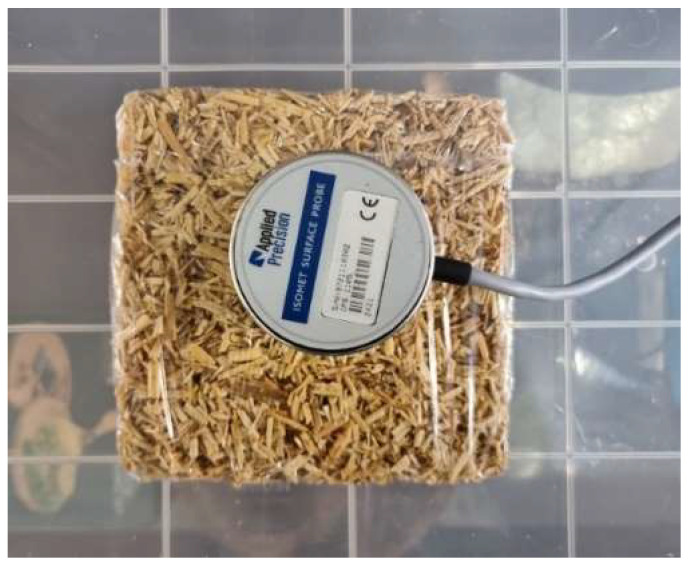
Measuring thermal conductivity with a disk probe.

**Figure 6:**
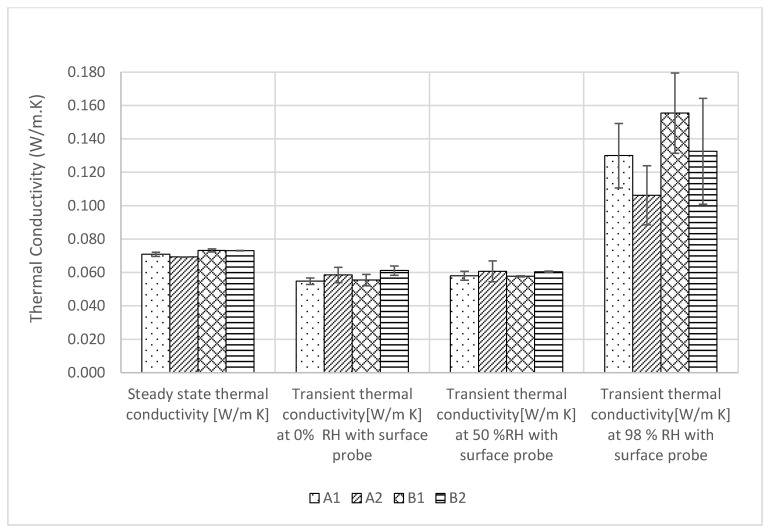
Thermal conductivity results.

**Figure 7:**
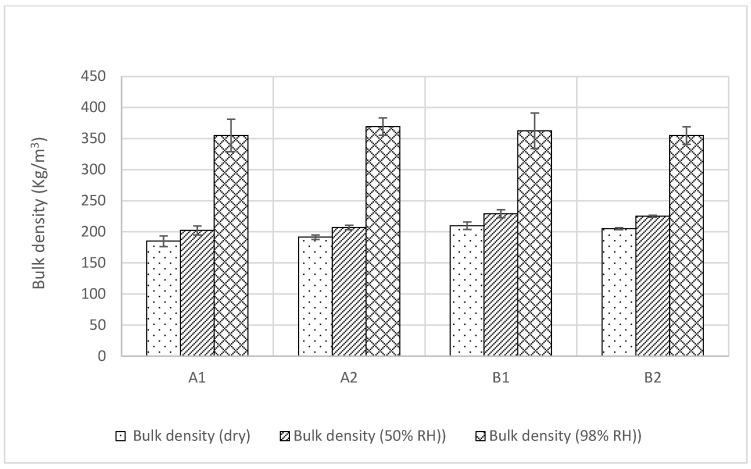
Bulk density and apparent bulk density of the samples.

The thermal conductivity of the samples was initially measured by the steady state method in a FOX 600 heat flow meter (TA Instruments, Cheshire, UK) at an ambient room condition of 23 ± 5 °C temperature and 50 ± 10% relative humidity. It was observed that all insulations have a similar thermal conductivity of 0.7 W/m.K, with negligible standard deviations. However, the transient measurements (Figure 5) using the Isomet Thermal Analyzer (Applied Precisions Ltd, Rača, Slovakia) show an average thermal conductivity value of 0.6 W/m.K at 0%, 50% relative humidity, and 23 °C temperature, which agrees with the value determined by Pundiene et al. [45]. The reduced thermal conductivity value recorded by the surface probe, compared to the values obtained in the heat flow meter, was plausibly caused by the air resistance between the surface of the specimen and that of the probe and the anisotropic nature of the insulation. The ASTM Committee C16 observes that transient methods can have a measurement precision of between ±10 and ±15% for rock and soil [38]. The current values show about the same trend. The transient method is more importantly used for measuring moisture-dependent thermal conductivity as it is difficult to do the same in a steady state heat flow meter due to the moisture migration induced by the temperature gradient across the depth of the samples. The transient measurements (Figure 5) show that, at 98% relative humidity and 23 °C temperature, the thermal conductivity of A1, A2, B1, B2 increased by 137%, 81%, 180%, and 117%, respectively, compared to their thermal conductivity values at 0% and 50% relative humidity (Figure 6). It was also observed that, at 98% RH, the apparent bulk density of A1, A2, B1, and B2 increased by 92%, 93%, 73%, and 73%, respectively, from the respective densities at 0% RH (Figure 7). The thermal conductivity of liquid water at 23 °C temperature is 0.056 W/m. K. It can be further observed in Figure 6 that at up to 50% relative humidity, moisture adsorption was about 20% in the insulation and that, according to the adsorption theory, they were mostly bonded to the internal surface as hydroxyl and thus not impact the thermal conductivity. However, at 98% relative humidity, condensation occurs in micropores and due to the liquid state of water, thermal conductivity increases.

Equations (6)–(8) were used to determine the moisture conversion functions (F_m_) and factors (f_u_ and f_ψ_) in relation to the moisture-dependent thermal conductivity changes from 0% to 98% relative humidity at 23 °C temperature (Table 5 and Table 6) following British Standard 10456:2007 [46]. Among other bio-based materials, the fitted f_ψ_ values correspond with the f_ψ_ value of expanded cork [46].

### 3.3. Vapour Diffusion Resistance Factor

**Figure 8:**
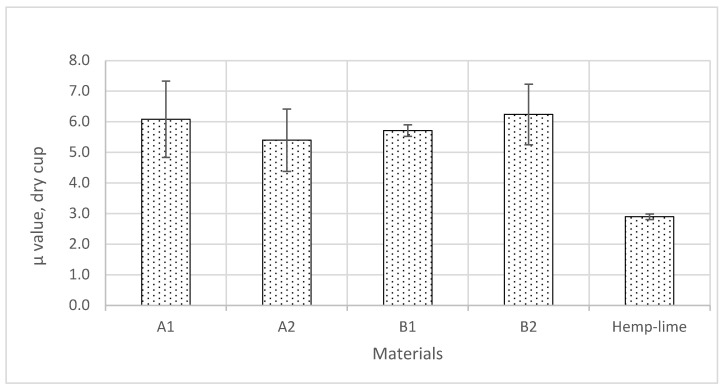
Vapour diffusion resistance factors (μ value, dry cup) of the samples.

The results of the vapour diffusion resistance factor tests, determined by the dry cup method, show that the average dry cup values vary between 5.4 and 6.2 (Figure 8). While the 10.7% variation in the highest and the lowest values is not significant, the average dry cup value of hemp–shive insulation is about double the value of hemp–lime insulation, which has a dry cup vapour diffusion resistance factor of 2.9 [11]. Within the narrow range of 10.7% difference, B2 exhibits the highest vapour diffusion resistance factor and A2 the lowest. Furthermore, high standard deviations can also be observed in some instances. The variations in the vapour diffusion values are observed plausibly due to the surface resistance of the materials. Furthermore, the hotwire anemometer readings show that the air velocity over the samples can vary between 0.1 and 0.7 m/s. It is difficult to maintain a steady-surface film resistance whilst running the experiment in the climate chamber since the placements of the samples varied both horizontally and vertically. However, the positions of the samples were swapped every 24 h to mitigate the effect of varying air velocity. Furthermore, another possible reason could be the tortuosity of the materials. Tortuosity is a function of pore size, pore distribution, and the degree and shape of pore connections.

### 3.4. Water Absorption Coefficient

**Figure 9:**
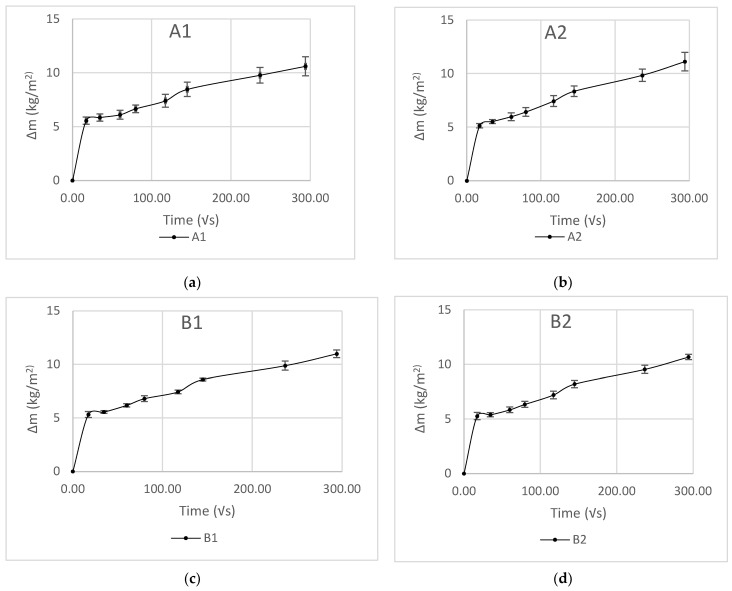
Water absorption kinetics of samples A1 (**a**), A2 (**b**), B1 (**c**), and B2 (**d**).

**Figure 10:**
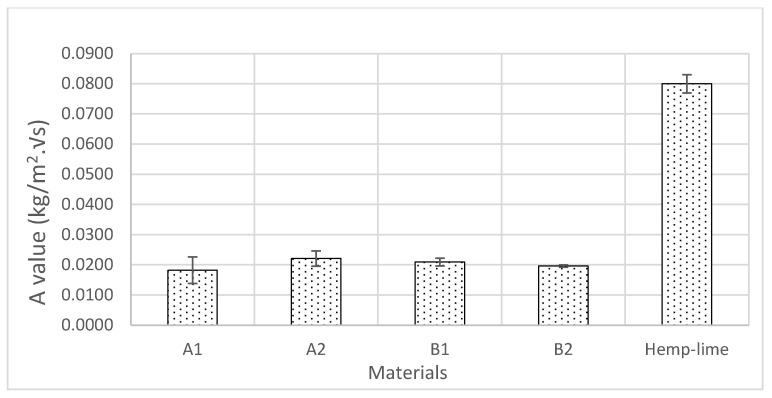
A value of samples A1, A2, B1, and B2 compared to hemp–lime.

**Figure 11:**
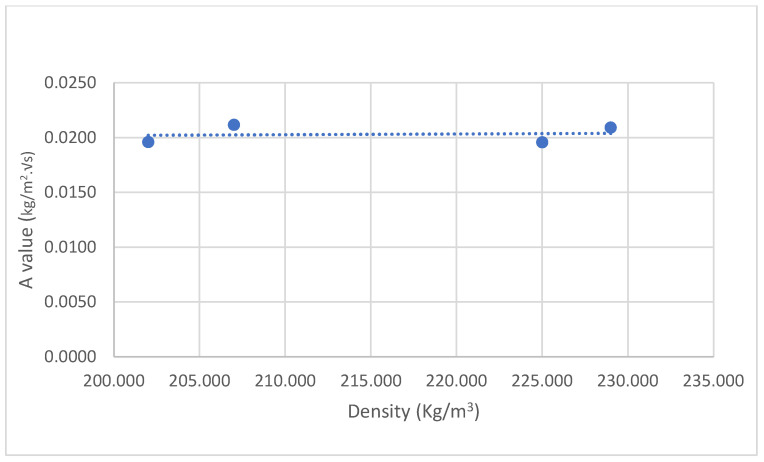
No strong linear relationship is observed between density and A value.

It was observed during the water absorption test of the samples that after a short period of stabilisation, the scatter pot of ∆m against √s showed a linear trend (Figure 9). Accordingly, following the British Standard 15148 [40], straight lines were drawn through the values and extended to intercept with the vertical axis, and the A values were determined using Equation (12) (Figure 10). No significant variations in the A value of A1, A2, B1, and B2 were observed. A1 demonstrated the highest standard deviation, which may have been due to the nature of the distribution of the binder in the matrix and the direction of the fibres. However, the water absorption values of the novel hemp–shive insulations were about one third of the water absorption values of hemp–lime insulations [47] and about half the value of hemp fibre insulations [44]. The findings agree with the observation by Gradinaru et al. [48] that the application of sodium silicate can reduce water absorption in bio-based materials. However, the effect of adding tributyl ester to sodium silicate on liquid water resistance is not evident in the water absorption behaviour of A2, while B2 exhibited a 6% decrease in A value compared to B1. A higher binder ratio and corresponding increase in tributyl ester in the mix of B2 could have caused the reduced A value. Furthermore, Figure 11 shows that the density of the samples did not influence the changes in A values significantly.

### 3.5. Porosity

**Figure 12:**
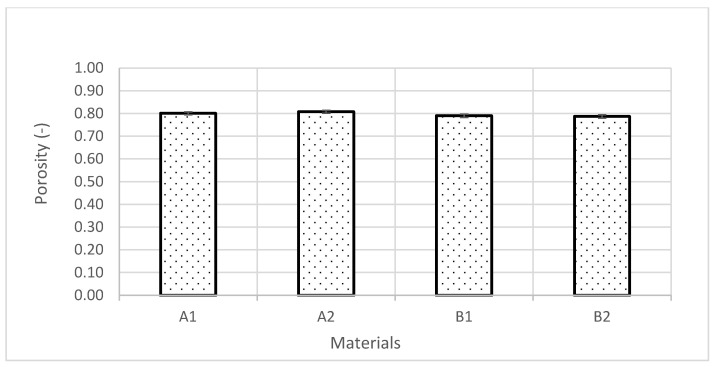
Porosity of samples A1, A2, B1, and B2.

Jiang et al. experimentally and numerically determined the following four absolute density values of hemp–shive [49]: 965 kg/m^3^, 977 kg/m^3^, 1023 kg/m^3^, and 1034 kg/m^3^. Using the absolute density values and applying Equation (14), the porosity of the novel insulation materials was determined (Figure 12). The results agree with the porosity values of wood fibre insulations of similar densities [50]. Furthermore, the value is comparable to the porosity value of 0.73 of hemp–lime that was determined by the mercury intrusion porosimetry method.

### 3.6. Hygrothermal Whole-Building Simulation

The whole-building hygrothermal simulation was carried out for one year following the methodology stated in Section 2.2.6 and using the data provided in Table 2, Table 3 and Table 4.

**Figure 13:**
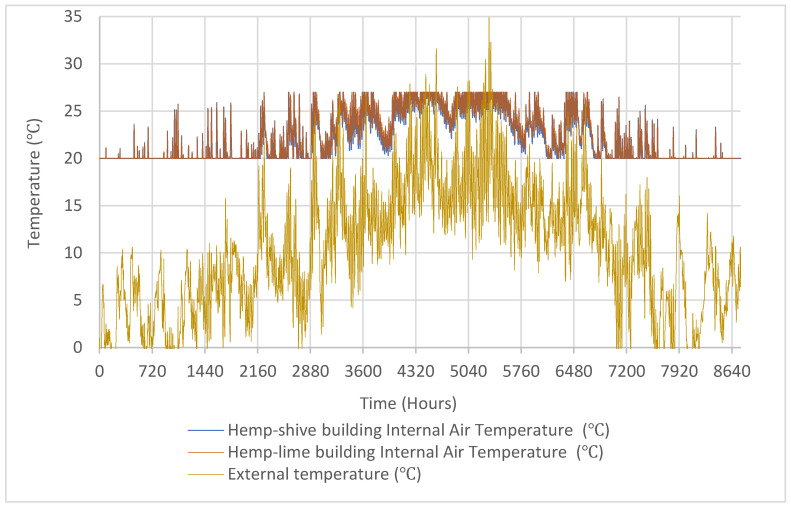
Outdoor and indoor relative humidity in the hemp–shive and hemp–lime buildings.

**Figure 14:**
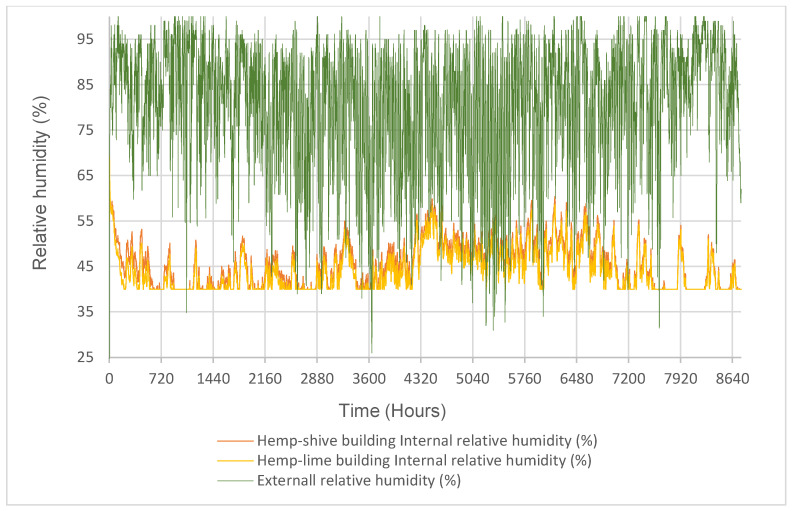
Outdoor and indoor temperature in the hemp–shive and hemp-line buildings.

**Figure 15:**
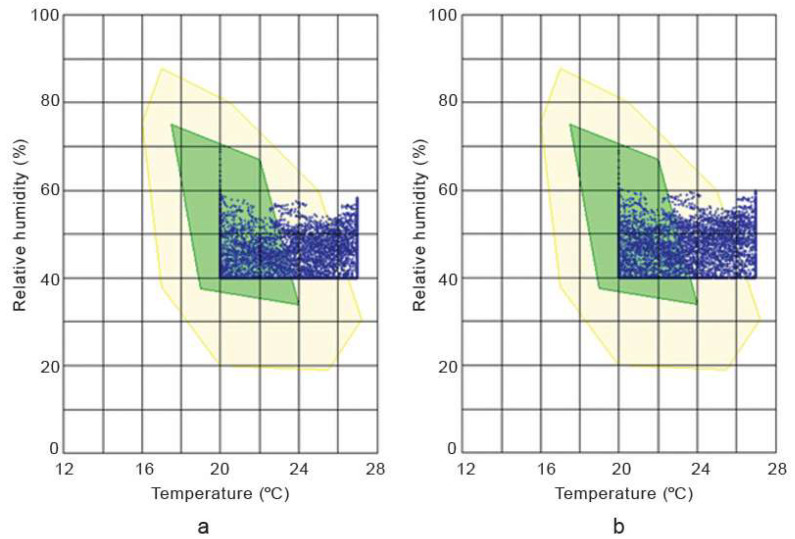
Thermal comfort conditions in (**a**) and hemp–lime (**b**) hemp–shive building. Green colour indicates ‘comfortable’ and yellow colour indicates ‘still comfortable’. The blue data points represent the relationship between temperature and realtive humidity.

**Figure 16:**
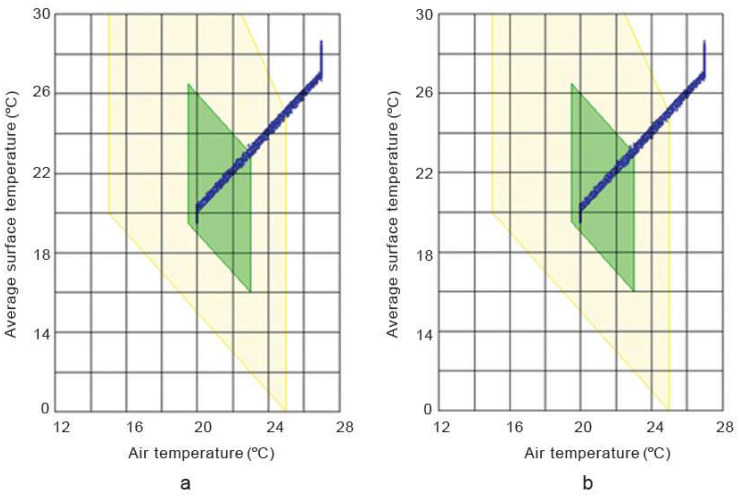
Surface temperature and indoor air temperature in (**a**) hemp–lime and (**b**) hemp–shive buildings. Green colour indicates ‘comfortable’ and yellow colour indicates ‘still comfortable’. The blue data points represent the relationship between air temperature and average surface temperature.

The hygrothermal simulation results in Figure 13 and Figure 14 show that the internal temperature and relative humidity trends in the hemp–lime and hemp–shive buildings do not show any significant difference provided that the temperature and relative humidity setpoints were 20 °C and 40% RH, respectively. A similar trend is observed in Figure 15 in terms of indoor thermal comfort. Figure 16 shows that, for both buildings, the difference between the surface and the indoor air temperature is less than 1 °C most of the time, which is also a thermal comfort requirement.

However, Table 7 reveals that the annual energy demand and annual heating demand in the hemp–shive building is 4.4% and 7.2% higher than those in the hemp–lime building, respectively. This may be because of the higher volumetric heat capacity of hemp–lime. In contrast, the annual cooling demand and humidity load in the hemp–lime building is 8.3% and 45.9% higher than those in the hemp–shive building, respectively. It is plausible that the high moisture buffer capacity of hemp–shive contributed to the lesser internal moisture load in the hemp–shive building. In terms of thermal comfort (Table 7), there is no significant difference between the two buildings.

**Figure 17:**
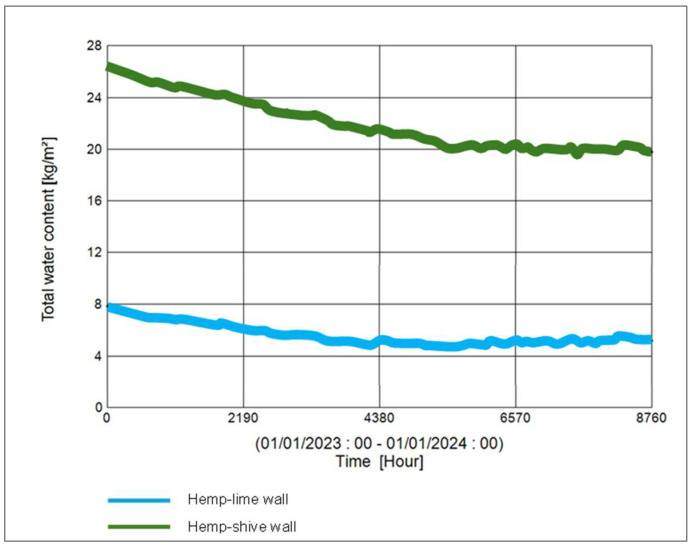
Total water content in hemp–shive and hemp–lime walls.

**Figure 18:**
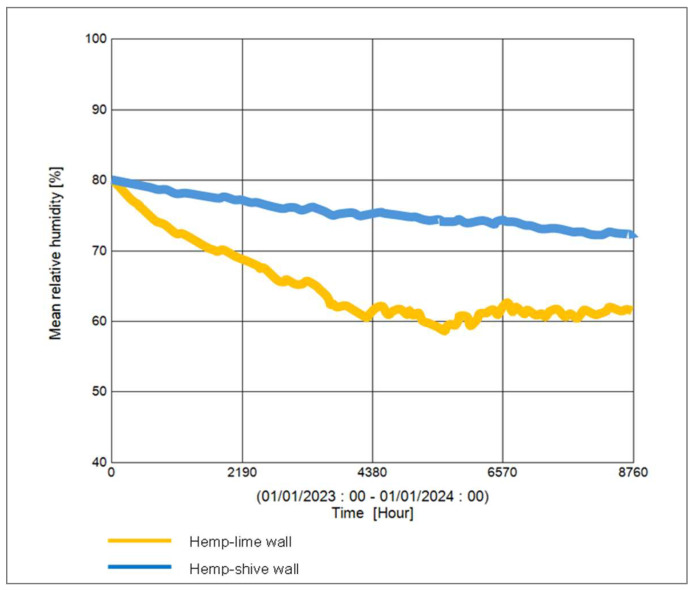
Relative humidity in hemp–shive and hemp–lime walls.

In terms of moisture management with the wall, it can be observed that (Figure 17) the total normalised water content in the hemp–shive wall is about 4 times higher than that in the hemp–lime wall; although, the drying rate is higher in case of the hemp–shive wall. A closer look at the adsorption isotherms reveals that the higher water content in the hemp–shive is caused by its higher adsorption via monolayer and multi-layer sorption (Figure 3). Figure 18 shows that the relative humidity in the hemp–lime stabilises to about 60%, while the relative humidity in hemp–shive is more than 70% during the last quarter of the year. More than 70% RH is concerning in terms of the possibility of mould growth. It can be concluded that there is no marked difference in the performance of hemp–shive and hemp–lime buildings in terms of their in-built hygrothermal performance. However, within the walls the relative humidity of hemp–shive is higher.

## 4. Conclusions

The primary aim of this paper was to assess the hygrothermal performance of four variants (A1, A2, B1, B2) of a novel hemp–shive insulation and to compare their performance with a novel hemp–lime insulation developed during the Hempsec project. The secondary aim was to compare the hygrothermal characters of A1, A2, B1, and B2 insulations. The insulations were experimentally characterised in terms of density, adsorption isotherm, water vapour diffusion resistance factor, and water absorption coefficient. The porosity values of the hemp–shive insulations were numerically determined. The thermal conductivity of the hemp–shive insulations at the ambient condition was measured with both steady-state and transient thermal conductivity measuring devices, while moisture-dependent thermal conductivity was measured with the transient device. Moisture-dependent thermal conductivity values were determined at the equilibrium moisture contents (EMCs) of 0%, 50%, and 98% relative humidity (RH) and 23 °C temperature. It was observed that there is no significant variation in the thermal conductivity of hemp–shive insulations between the EMCs at 0% RH and 50% RH, but there is a substantial increase in thermal conductivity when the insulation material reaches the EMC at 98% RH. The dry thermal conductivity values of hemp–shive and hemp–lime insulations were similar, although the values serve as a reference only since 0% RH at 23 °C is not a natural condition.

The adsorption isotherms of hemp–shive insulations were determined at the following RH steps: 0%, 20%, 50%, 70%, 90%, and 98% at a constant temperature of 23 °C. All isotherms show the International Union of Pure and Applied Chemistry (IUPAC) Type II pattern, meaning that moisture adsorption increases exponentially from 70% RH with multilayer sorption. At 98% RH, the moisture adsorption capacity of the hemp–shive insulation is 4-to-5 times higher than that of the hemp–lime insulation. As such, there is a likelihood that hemp–shive insulations will act as a better moisture buffering material than hemp–lime.

The dry-cup vapour diffusion tests show that the materials’ vapour diffusion resistance factor (µ value) varies between 5 and 6, which is about double the µ value of hemp–lime insulation. Combined with high adsorption capacity, hemp–shive insulations are likely to act as a better hygric mass. The water absorption test shows that the water absorption coefficients (a value) vary between 0.018 and 0.022 kg/m^2^√s, providing 4-to-5 times higher water absorption resistance than that of hemp–lime insulation. It is a significant achievement, as one of the objectives was to reduce liquid water absorption by hemp–shive. Numerically determined porosity values agree with the values of wood-based insulation materials of similar density.

Finally, all material data were used as input data in WUFI 3D software (WUFI@Plus V.3.2.0.1) to compare the in-built hygrothermal and energy performance of the hemp–shive materials with the high-performance hemp–lime insulation material via numerical hygrothermal simulations. It was found that the novel hemp–shive insulation materials perform at a similar level to the hemp–lime insulation materials in terms of heating and cooling energy demand but required less energy for humidification. This implies that hemp–shive possesses superior hygric capacity. However, it was also observed that the relative humidity inside the hemp–shive wall was always more than 70%, which may induce mould growth inside the wall.

While hemp–shive insulation offers the possibility of being a fully bio-degradable composite with lesser embodied energy due to its lower binder to hemp–shive ratio, it will likely be another variant of hemp-based insulation rather than a replacement for hemp–lime since hemp–lime is suitable for in situ casting whereas the novel hemp–shive insulation is suitable for producing factory-based prefabricated panels.

## Figures and Tables

**Table 1:** Material density and types.

Name	Dimension (±2 mm)	Buk Density (kg/m^3^)	Constituents	Binder-to-Shive Ratio by Mass	Addition of Tributyl Ester
A1 (3 samples)	150 × 150 × 50	202	Hemp–shive, binder	1.5:1	N/A
A2 (3 samples)	150 × 150 × 50	207	Hemp–shive, binder	1.5:1	10% by mass added to the binder
B1 (3 samples)	150 × 150 × 50	229	Hemp–shive, binder	1.75:1	N/A
B2 (3 samples)	150 × 150 × 50	225	Hemp–shive, binder	1.75:1	10% by mass added to the binder

**Table 2:** Building and comfort parameters.

	Layers	Material	Thermal Conductivity (W/m.K)	Thickness (mm)	U Value of the Assembly (W/m^2^.K)
Hemp–lime wall assembly	Layer 1 (outside)	Hydraulic lime	0.8	15	0.15
	Layer 2	Hemp–lime	0.07	450	
	Layer 3 (inside)	Hydraulic lime	0.8	15	
Hemp–shive wall assembly	Layer 1 (outside)	Hydraulic lime	0.8	15	0.15
	Layer 2	Hemp–shive	0.063	408	
	Layer 3 (inside)	Hydraulic lime	0.8	15	
Roof assembly	Layer 1 (outside)	Wood fibre board	0.044	400	0.11
	Layer 2	Gypsum plaster	0.2	10	
Floor assembly	Layer 1 (top)	Concrete screed	1.6	150	0.15
	Layer 2	Mineral wool	0.035	224	
	Layer 3	Vapour retarder	2.3	1	
	Layer 4	Concrete	1.6	40	
Window (Solar transmission factor: 0.59)	Layer 1 (outside)	Glass	-	4	0.8
	Layer 2	Argon (90%)	-	16	
	Layer 3	Glass	-	4	
	Layer 4	Argon	-	16	
	Layer 5 (inside)	Glass (90%)	-	4	

**Table 3:** Building comfort parameters.

Temperature Setpoint (°C)	Relative Humidity Setpoint (%)	Ventilation Rate (1/h)	Infiltration Rate (1/h)	Initial CO_2_ Concentration (ppmv)
20	40	0.5	0.6	3000

**Table 4:** Material characterisation data of hemp–shive and hemp–lime insulation.

	Bulk Density at 23 °C and 0% RH (kg/m^3^)	Thermal Conductivity at 0% RH (W/m.K)	Thermal Conductivity at 23 °C and 95% RH (W/m.K)	Thermal Conductivity at 23 °C and 98% RH (W/m.K)	Specific Heat Capacity (J/kg.°C)	Porosity (-)	Vapour Diffusion Resistance Factor, Dry Cup (-)	Water Absorption Coefficient (kg/m^2^.√s)
Hemp–shive	185	0.063	-	0.13	1880	0.8	6.1	0.018
Hemp–lime	240	0.07	0.125	-	2000	0.85	2.9	0.08

**Table 5:** Conversion factors mass by mass for determining moisture-dependent thermal conductivity at 98% RH.

Sample	F_m_	e	f_u_	u_2_	u_1_	λ_2_
A1	2.37	2.718	0.865	1.00	0	0.130
A2	1.80	2.718	0.69	0.85	0	0.106
B1	2.79	2.718	1.415	0.73	0	0.155
B2	2.16	2.718	1.06	0.73	0	0.133

**Table 6:** Conversion factors volume by volume for determining moisture-dependent thermal conductivity at 98% RH.

Sample	F_m_	e	f_ψ_	ψ_2_	ψ_1_	λ_2_
A1	2.385	2.718	4.700	0.185	0	0.130
A2	1.830	2.718	3.700	0.163	0	0.106
B1	2.776	2.718	6.700	0.152	0	0.155
B2	2.179	2.718	5.200	0.150	0	0.133

**Table 7:** The heating, cooling, and humidification loads of hemp–shive and hemp–lime buildings.

	Annual Heating and Cooling Demand (kWh)	Annual Heating Demand (kWh)	Annual Cooling Demand (kWh)	Humidification (Kg)
Hemp–shive building	2690.1	2239.6	450.5	194.4
Hemp–lime building	2577.4	2089.5	487.9	283.7

## Data Availability

Data are contained within the article.

## References

[1] Standard, UK Net Zero Carbon Building “Uk Net Zero Carbon Building Standard. Technical Update & Consultation”. 14 June 2023. https://www.nzcbuildings.co.uk/.

[2] Akhimien N.G., Latif E., Hou S.S. (2021). Application of Circular Economy Principles in Buildings: A Systematic Review. J. Build. Eng..

[3] Khadim N., Agliata R., Thaheem M.J., Mollo L. (2023). Whole Building Circularity Indicator: A Circular Economy Assessment Framework for Promoting Circularity and Sustainability in Buildings and Construction. J. Affect. Disord..

[4] European Commission In Focus: Energy Efficiency in Buildings. European Commission..

[5] Department for Business, Energy and Industrial Strategy (BEIS) (2022). UK Energy in Brief 2022.

[6] Envoys, Energy. Energy Tutorial: Energy and Sustainability. What’s Energy Used For?. http://www.energyenvoys.org.uk/.

[7] Stefanowski B., Curling S., Ormondroyd G. (2017). Assessment of Lignocellulosic Nut Wastes as an Absorbent for Gaseous Formaldehyde. Ind. Crops Prod..

[8] van der Werf H.M.G. (1991). Agronomy and Crop Physiology of Fibre Hemp: A Literature Review.

[9] Reddy B.V., Jagadish K.S. (2003). Embodied Energy of Common and Alternative Building Materials and Technologies. Energy Build..

[10] Lecompte T., Levasseur A., Maxime D. (2017). Lime and Hemp Concrete LCA: A Dynamic Approach of GHG Emissions and Capture. Acad. J. Civ. Eng..

[11] Latif E., Lawrence M., Shea A., Walker P. (2015). Moisture Buffer Potential of Experimental Wall Assemblies Incorporating Formulated Hemp-Lime. J. Affect. Disord..

[12] Moletti C., Aversa P., Losini A., Dotelli G., Woloszyn M., Luprano V. (2023). Hygrothermal Behaviour of Hemp-Lime Walls: The Effect of Binder Carbonation over Time. J. Affect. Disord..

[13] Collet F., Pretot S. (2014). Thermal Conductivity of Hemp Concretes: Variation with Formulation, Density and Water Content. Constr. Build. Mater..

[14] Collet F., Pretot S. (2012). Experimental Investigation of Moisture Buffering Capacity of Sprayed Hemp Concrete. Constr. Build. Mater..

[15] Shea A., Lawrence M., Walker P. (2012). Hygrothermal Performance of an Experimental Hemp–Lime Building. Constr. Build. Mater..

[16] Rachel B., Woolley T. (2008). Hemp Lime Construction: A Guide to Building with Hemp Lime Composites.

[17] Andy S., Black D., Walker P., Building Research Establishment (2011). Hemp Lime: An Introduction to Low Impact Building Materials.

[18] Haik R., Bar-Nes G., Peled A., Meir I. (2020). Alternative Unfired Binders as Lime Replacement in Hemp Concrete. Constr. Build. Mater..

[19] Gacoin A., Li A. (2023). Optimal Composition of a Starch-Hemp Agro-Composite Materials. Constr. Build. Mater..

[20] Haik R., Peled A., Meir I. (2020). The Thermal Performance of Lime Hemp Concrete (LHC) with Alternative Binders. Energy Build..

[21] Bragaglia M., Mariani M., Sergi C., Sarasini F., Tirillò J., Nanni F. (2023). Polylactic Acid as Biobased Binder for the Production of 3D Printing Filaments for Ti6Al4V Alloy Manufacturing via Bound Metal Deposition. J. Mater. Res. Technol..

[22] Wennman M., Hellberg M., Svagan A.J., Hedenqvist M.S. (2023). A Biobased Binder of Emulsion Type That Provides Unique and Durable Wet Strength and Hydrophobicity to Paper and Nonwoven. Ind. Crops Prod..

[23] Wennman M., Pinon A.C., Svagan A.J., Hellberg M., Hedenqvist M.S. (2024). A Biobased Binder of Carboxymethyl Cellulose, Citric Acid, Chitosan and Wheat Gluten for Nonwoven and Paper. Carbohydr. Polym..

[24] Odiyi D., Sharif T., Choudhry R., Mallik S., Shah S. (2023). A Review of Advancements in Synthesis, Manufacturing and Properties of Environment Friendly Biobased Polyfurfuryl Alcohol Resin and Its Composites. Compos. Part B Eng..

[25] Laurichesse S., Avérous L. (2014). Chemical Modification of Lignins: Towards Biobased Polymers. Prog. Polym. Sci..

[26] Van Nieuwenhove I., Renders T., Lauwaert J., De Roo T., De Clercq J., Verberckmoes A. (2020). Biobased Resins Using Lignin and Glyoxal. ACS Sustain. Chem. Eng..

[27] Alsalman A., Assi L.N., Kareem R.S., Carter K., Ziehl P. (2021). Energy and CO_2_ Emission Assessments of Alkali-Activated Concrete and Ordinary Portland Cement Concrete: A Comparative Analysis of Different Grades of Concrete. Clean. Environ. Syst..

[28] Fawer M., Concannon M., Rieber W. (1999). Life Cycle Inventories for the Production of Sodium Silicates. Int. J. Life Cycle Assess..

[29] Hanafi M., Ekinci A., Aydin E. (2020). Triple-Binder-Stabilized Marine Deposit Clay for Better Sustainability. Sustainability.

[30] UN-HABITAT (1991). Energy for Building Improving Energy Efficiency in Construction and in the Production of Building Materials in Developing Countries.

[31] Andreola F., Barbieri L., Lancellotti I. (2019). The Environmental Friendly Route to Obtain Sodium Silicate Solution from Rice Husk Ash: A Comparative Study with Commercial Silicates Deflocculating Agents. Waste Biomass-Valorization.

[32] Handayani L., Aprilia S., Abdullah, Rahmawati C., Aulia T.B., Ludvig P., Ahmad J. (2022). Sodium Silicate from Rice Husk Ash and Their Effects as Geopolymer Cement. Polymers.

[33] European Commission Market Development of a Bio-Based Pre-Fabricated Construction System Which Significantly Reduces Both Embodied Carbon and in-Use Energy Consumption (Hempsec). https://ec.europa.eu/info/funding-tenders/opportunities/portal/screen/how-to-participate/org-details/952866503/project/332972/program/31005240/details.

[34] Yu Z., Wan Y., Qin Y., Jiang Q., Guan J.-P., Cheng X.-W., Wang X., Ouyang S., Qu X., Zhu Z. (2023). High Fire Safety Thermal Protective Composite Aerogel with Efficient Thermal Insulation and Reversible Fire Warning Performance for Firefighting Clothing. Chem. Eng. J..

[35] (2021). Hygrothermal Performance of Building Materials and Products—Determination of Hygroscopic Sorption Properties.

[36] (2015). Standard Test Method for Steady-State Thermal Transmission Properties by Means of the Heat Flow Meter Apparatus.

[37] (2019). Thermal Insulation. Determination of Steady-State Thermal Resistance and Related Properties Heat Flow Meter Apparatus.

[38] (2008). Standard Test Method for Determination of Thermal Conductivity of Soil and Soft Rock by Thermal Needle Probe Procedure D-5334-08.

[39] (2013). Thermal Insulating Products for Building Applications. Determination of Water Vapour Transmission Properties.

[40] (2016). Hygrothermal Performance of Building Materials and Products—Determination of Water Absorption Coefficient by Partial Immersion.

[41] Steve C. (2015). Petrophysics: A Practical Guide.

[42] Ron J., Neymark J. (1998). The Bestest Method for Evaluating and Diagnosing Building Energy Software.

[43] Latif E., Bevan R., Woolley T. (2019). Thermal Insulation Materials for Building Applications.

[44] Latif E., Tucker S., Ciupala M.A., Wijeyesekera D.C., Newport D. (2014). Hygric Properties of Hemp Bio-Insulations with Differing Compositions. Constr. Build. Mater..

[45] Pundiene I., Vitola L., Pranckeviciene J., Bajare D. (2022). Hemp Shive-Based Bio-Composites Bounded by Potato Starch Binder: The Roles of Aggregate Particle Size and Aspect Ratio. J. Ecol. Eng..

[46] (2007). Building Materials and Products. Hygrothermal Properties. Tabulated Design Values and Procedures for Determining Declared and Design Thermal Value.

[47] Ruus A., Koosapoeg T., Pau M., Kalamees T., Põldaru M. (2021). Influence of Production on Hemp Concrete Hygrothermal Properties: Sorption, Water Vapour Permeability and Water Absorption. J. Phys. Conf. Ser..

[48] Gradinaru C.M., Barbuta M., Ciocan V., Antohie E., Babor D. (2018). The Effects of Sodium Silicate on Corn Cob Aggregates and on the Concrete Obtained with These Agricultural Waste. IOP Conf. Ser. Mater. Sci. Eng..

[49] Jiang Y., Lawrence M., Ansell M.P., Hussain A. (2018). Cell Wall Microstructure, Pore Size Distribution and Absolute Density of Hemp Shiv. R. Soc. Open Sci..

[50] De Ligne L., Van Acker J., Baetens J.M., Omar S., De Baets B., Thygesen L.G., Bulcke J.V.D., Thybring E.E. (2022). Moisture Dynamics of Wood-Based Panels and Wood Fibre Insulation Materials. Front. Plant Sci..

